# Supplementary material for: Evolution of European Resuscitation and End-of-Life Practices from 2015 to 2019: A Survey-Based Comparative Evaluation
Source: J Clin Med. 2022 Jul 11;11(14):4005. doi: 10.3390/jcm11144005 (PMC9316602; doi:10.3390/jcm11144005)
Supplement: Supplementary file 1 [file jcm-11-04005-s001.zip › jcm-1761861-supplementary.pdf]

ONLINE SUPPLEMENT TO:
EVOLUTION OF EUROPEAN RESUSCITATION AND END-OF-LIFE PRACTICES FROM 2015 TO 2019: A SURVEY-BASED COMPARATIVE EVALUATION.
SUPPLEMENTAL METHODS
Ethics Committee Approval

The study protocol was approved by the Regional Ethics and Scientific Committee of Evaggelismos General Hospital of Athens, the Athens Eye Clinic, and the Athens Polyclinic (Approval No. 447/29/7/2019). The approval was used to support the conduct of the survey study in 32 European countries and Turkey. The 32 European countries are listed as follows: Austria, Belarus, Belgium, Bosnia & Herzegovina, Croatia, Cyprus, Czech Republic, Denmark, Finland, France, Germany, Greece, Hungary, Iceland, Italy, Ireland, Luxembourg, Malta, Norway, Poland, Portugal, Republic of Northern Macedonia, Romania, Russian Federation, Serbia, Slovakia, Slovenia, Spain, Sweden, Switzerland, The Netherlands, and the United Kingdom. Study participant eligibility criteria are detailed in the Methods section of the main paper.

The informed consent procedure

The following text for potential study participants was presented as part of the study protocol, as well as in informed consent format attached to an invitation email message:

According to the Helsinki declaration, participation in research has the essential prerequisite of free and voluntary informed consent of the participant.

According to Regulation 679/2016 {or General Data Protection regulation (GDPR) of the European Parliament and of the Council}, *""consent" of the data subject means any freely given, specific, informed and unambiguous indication of the data subject's wishes by which he or she, by a statement or by a clear affirmative action, signifies agreement to the processing of personal data relating to him or her."*

Participation in the current survey study means that you accept to share your knowledge on practices / decisions related to end-of-life care (domain A), on access to best-quality care (domain B), on death diagnosis and organ donation (domain C), and on emergency care organization (domain D) in your country.

According to GDPR, *"pseudonymization means the processing of personal data in such a manner that the personal data can no longer be attributed to a specific data subject without the use of additional information, provided that such additional information is kept separately and is subject to technical and organizational measures to ensure that the personal data are not attributed to an identified or identifiable natural person."*

A set of 50 consecutive random numbers (range, 50-99) will be generated using "Research Randomizer (www.randomizer.org). Each one of the aforementioned numbers will be assigned to a European country (country code) drawn according to alphabetic order from the list of participating European countries. Next to the 2 digits of the country code, a third digit corresponding to your temporal order of participation relative to other participants from your country; your temporal order will be determined according to the exact time of completion of the study questionnaire. This will form your personal, 3-digit, code.

Data on questionnaire domains will be stored next to your personal code in Microsoft Excel format. This electronic Masterfile will be stored onto the password-protected hard disk of the principal investigator's personal computer. In addition, next to your personal code, we will enter the following personal data 1) name; professional qualification(s) (e.g. physician, nurse, paramedic, other); 2) highest academic or professional rank (e.g. professor, lead clinician, lead nurse, etc.); 3) professional occupation - more than one possible (e.g. academic teaching, academic research; hospital emergency department, intensive care unit, emergency medical service, etc.); and 4) years of professional experience as healthcare provider, academic, or both. Subsequently, personal codes and names will be copied in a second Excel file, the Decoding

File. Finally, all names will be removed from the Masterfile, whereas the personal, 3-digit codes will be retained. Consequently, the Masterfile will fulfill the criterion of *"pseudonymization."*

The Masterfile data will be used for statistical analyses aimed at testing the study hypotheses. In this context, and within the next 3-4 months, we will resend to you the data corresponding to your responses in Excel format so that you may be able to confirm their accuracy. In addition, we will also send to you the data corresponding to other respondents from your country so that you may be able to state whether you agree with their responses. In the event of disagreement(s) we may request that you attempt to reach consensus with the other respondents from your country. However, please note that the reaching of such consensus should not in anyway be considered as "mandatory".

According to the above, we request your free and voluntary consent for

1) Collection and storing of the above-mentioned data under password protection;

and

2) Inclusion of the above-mentioned data (at least in part) in an Acknowledgment section of a published article reporting on the results of the survey study.

As essential prerequisites for consent validity we ask you to respond to the following questions:

A) Have you read / understood the above-presented information?
B) Have you had adequate time to consider granting your consent for the use of your personal data?
C) If you asked any questions about any aspect of the survey study, did you receive satisfactory replies from the study investigator?
D) Do you understand that you may withdraw your consent at any time until the publication of the survey study results?
E) Do you understand that after publication of the study results and dissemination of your personal data, it may become not possible to reverse such dissemination?
F) Do you agree with the aforementioned procedure of pseudonymization and subsequent dissemination of your personal data?

To gain electronic access to the survey questions through the survey's website [https://www.surveymonkey.com/r/22YQS7R], study participants had to first click on an "Accept" button for all the above "Terms and Conditions". Acceptance of "Terms and Conditions" was considered as positive response to all the above-mentioned consent-related requests and questions.

Compliance with the Checklist for Reporting Results of Internet E-surveys (CHERRIES).

Survey design: Inclusion criteria for study participants and minimum target enrollment for each country are detailed in the second to fourth paragraph of the Methods section (pages 3 and 4 of the main paper).

Institutional Review Board (IRB) approval and informed consent process: Information about the IRB that approved the study is provided in the first paragraph of the Methods section (page 4 of the main paper). The informed consent process is detailed in the preceding section of the current supplement. Study participants were aware of the anticipated duration of the survey (i.e. approximately 20-30 min) as well as of the anticipated duration of the study *per se*, as mentioned in the prospectively registered protocol (Clinicaltrials.gov Identifier: NCT04078815). In addition, as detailed in the preceding section of the current supplement, participants were fully informed about which data need to be stored, the relevant storage safety features, the anticipated duration of the storage, and the intended, final disposition of the data. The main safety feature for participants' personal data protection comprised encoding and data storage onto a password-protected hard disk; the pertinent files were accessible solely by the current study's principal investigator (SDM).

Development and testing: Due to the current study's specific objectives (i.e. comparison of end-of-life practices between 2019 and 2015; see also Introduction of the main paper), the current questionnaire included precisely the same questions to those used in a previously published survey study [1]. Additional questions on end-of-life practices / decisions (n=2), and emergency care organization (n=8) were added on the basis of investigator consensus; these questions were not included in the 2019 vs. 2015 comparative analysis (see also footnote of Table 1 of the main paper). The questionnaire was tested by the current paper's first (SDM) and last author (LB) before granting access to study participants.
Recruitment and description of study participants having access to the questionnaire: This was a personal e-mail invitation-based, closed survey with specific participant inclusion criteria pertaining to nationally and/or internationally recognized, specific, clinical and/or research expertise in the field of resuscitation and end-of-life care (see also second paragraph of Methods in pages 3 and 4 of the main paper).
Survey administration: As stated in the first paragraph of the Methods (page 5 of the main paper), this was a web-based survey accessible through a Survey Monkey link. Survey Monkey is a well-established website providing access to software enabling survey creation.
Item category: This was a mandatory survey and responses and participants were expected to respond to all of its questions. No incentives were offered. The time-frames of data collection periods are detailed in the fourth paragraph of the Methods (page 5 of the main paper), in Figure 1 of the main paper, and in the second paragraph of the Statistical analysis (page 8 of the main paper – data was collected for the purpose of an un-prespecified, exploratory analysis). Survey items were not randomized and no adaptive questioning was used. The number of survey items (i.e. sets of questions organized in domain subsections - presented in Table 1 of the main paper) per page was within 0.3-2, depending on the number of questions per item, which ranged from 1 to 8 (see also Table 1 of the main paper). The questionnaire was distributed over 15 pages. Completeness checks were possible before the completion of the survey by returning on a previous page. As stated in the fourth paragraph of the Methods (page 5 of the main paper) respondents had to choose either among 4 options, i.e. *never*, *sometimes*, *usually* and *always* or between *no* and *yes*. Selection of only one response was enforced. The option of "not applicable" was provided for the response to the second question of subsection D2, and the first question of subsection D6 (see also Table 1 of the main paper). Lastly, as stated in the fourth paragraph of the Methods (page 5 of the main paper, respondents were asked to reconfirm their answers approximately 3 months after the initial email invitation; the questions and their answers were concurrently emailed to them in excel format.
Response rates: Determination of unique site-visiting invitees was based on their internal protocol (IP) addresses. All 85 visiting invitees who provided consent for participation (see preceding subsection and first paragraph of the main paper's Results section) viewed and participated in/completed the survey (view/participation/completion rate: 100%). However, just 5 out of the 85 participants initially responded to the total of the survey questions (initial completeness rate: 5.9%); these respondents originated from Croatia (n=1), Greece (n=2), Hungary (n=1), and Romania (n=1).
Preventing multiple entries from the same individual: In case of a duplicate entry from the same IP address, the most recent one was to be retained for the analysis. However, there were no duplicate entries.
Analysis: Only completed questionnaires were analyzed. The methodology used to increase questionnaire completion and resolution of discrepancies between respondents from the same country is detailed in the fourth paragraph of the Methods (page 5 of the main paper); the corresponding results concerning the provision of initially missing responses and discrepancies' resolution through consensus are reported in the first paragraph of the Results (page 9 of the main paper). We did not measure the time respondents needed to fill in the

questionnaire. Furthermore, we did not apply any statistical correction to adjust for the possibly ″nonrepresentative″ sample.

Statistical Analysis – additional details on linear regression

As reported in the supplement of our 2016 paper reporting the results of our 2015 survey [1], bivariate linear regression was performed to explore a possible association between domain A and domain D scores. The dependent variable was domain D score with and without the "new - 2019-only" questions (see also Statistical analysis of the main paper). Autocorrelation (i.e. the similarity between observations as a function of the time lag between them) was assessed with the Durbin-Watson test. Possible heteroscedasticity (i.e. the unequal variability of the dependent variable across the range of values of the independent variable) was assessed by visual inspection of the normal expected-observed probability plot of the regression standardized residual. Statistical significance was accepted at $P<0.05$. Analyses were performed with SPSS version 28.0 (IBM Corporation, Armonk, New York, United States).

SUPPLEMENTAL RESULTS

Table S1 displays country-specific initial numbers of discrepancies in responses (i.e. conflicting) and/or missing responses, and outcome of efforts to resolve such issues. Such efforts comprised 1) encouraging respondents to reach consensus on conflicting and/or missing responses (for countries with >1 respondents); or 2) requesting/encouraging additional effort from single, country-specific respondents to provide any initially missing responses.

Table S2 displays country-specific, Domain A to D scores for 2015 and 2019. In Domains A, B, and D, there were reported changes of ≥10 points (i.e. ≥24.4% of the maximum possible score) in 5/25 (20.0%), 4/25 (16.0%), and 2/25 (8.0%) countries, respectively. However, in Domains A and B, these large score changes had varying directions (i.e. 4 positive and 1 negative in A, and 3 negative and 1 positive in B), thereby resulting in relatively minor effects on the respective mean and median values. Table S2 also displays (in brackets) the 2019 Domain A and D scores after adding subscores of responses to questions not included in the 2015 questionnaire.

Tables S3 displays detailed results, including scores of responses to sets of questions concerning each one of the variables (e.g. advance directives, diagnostic criteria for death, etc.) included in the 2019 and 2015 questionnaires. Tables S4 and S5 display detailed results on countries with "low" and "high" 2015 domain A to D scores, respectively; "low" and "high" scores are defined in the Methods of the main paper.

Responses to questions included only in the 2019 survey are detailed below:

Domain A: Country-specific rate of positive responses was 60% and 24-52% for legal support and application of advance care planning (ACP) in various settings, respectively. Positive response rate was 80% and 76% for shared decision making for adults and children, respectively.

Domain D: Country-specific rate of positive responses was 1) 44-100% for dispatcher-assisted cardiopulmonary resuscitation (CPR) and guidance about compressions/ventilation; 2) 24-88% for specific quality features of prehospital (ambulance) care; 3) 48- 72% for educational programs in the field of ethics; 4) 20-44% for CPR training/certification; and 5) 12-36% for research without pre-enrollment consent. Lastly, positive response rate was 32% for the availability of automated external defibrillator data (e.g. cardiac rhythm analysis) in patient record, and 64% for the presence a trained rescuer alert system (e.g. text message or specific smartphone application).

Exploratory analyses

Bivariate linear regression analyses revealed significant associations between the 2019 domain A (independent variable) and domain D (dependent variable) scores either with (adjusted $r^2=0.35$; $P=0.001$; Figure S1A) or without (adjusted $r^2=0.43$; $P<0.001$; Figure S1B) taking into account the scores of responses to questions not included in the 2015 questionnaire [1]

(Table S2). This indicates that the 2019 Domain A score explained 35-43% of the variance of the 2019 domain D score. There were also strong linear relationships between the 2019 score for do-not-attempt CPR (DNACPR) and ACP (r^2=0.68, P <0.001; Figure S1C) and the 2019 score for advance directives and ACP (r^2=0.79, P <0.001; Figure S1D).

Table S6 displays results on coronavirus disease-19 related changes. Respondents from 15/25 countries (60.0%) reported "no change". Furthermore, respondents from 8/25 countries (32.0%) reported minor changes, resulting in either domain A, or domain B, or domain D score changes of -3 to +2 points; briefly, 3 countries reported that access to resuscitation care may now be affected by age, comorbidity, high risk presentation, health system overload / COVID-19-related lockdown, and deactivation of applications alerting rescuers/responders on the occurrence of sudden cardiac arrest; two countries reported discontinuation of family presence during resuscitation for adults and/or children; one country reported a reduction in organ transplantation procedures; one country reported initiation of in-hospital audit for CPR; one country reported that consultants are now obliged to state the DNACPR status of admitted patients, and that an in-hospital cardiac arrest registry has been started; finally, one country mentioned possible problems with the continuation of registry reporting of out-of-hospital and in-hospital cardiac arrest. Survey participants from 2/25 countries (8.0%) did not provide a COVID-19-related response (Table S6).

REFERENCES

1. Mentzelopoulos SD, Bossaert L, Raffay V, Askitopoulou H, Perkins GD, Greif R, Haywood K, Van de Voorde P, Xanthos T. A survey of key opinion leaders on ethical resuscitation practices in 31 European Countries. Resuscitation. 2016;100:11-7.

Table S1. Country-specific, initial number of discrepancies in responses and/or missing responses, and outcome of efforts to resolve these issues*.

Country	Respondents - No.	Domain A - No.	Domain B - No.	Domain C - No.	Domain D - No.	Sum - (%) of total†	Outcome of Efforts to resolve problems with responses*
Austria	3	7	9	4	36	56 (31.3)	Consensus achieved
Belarus	2	43	0	13	48	104 (58.1)	Consensus not achieved
Belgium	9	49	15	16	68	148 (82.7)	Consensus achieved
Croatia	1	0	0	0	0	0 (0.0)	No missing response
Cyprus	3	40	11	10	44	105 (58.7)	Consensus achieved
Czech Republic	2	15	4	4	22	45 (25.1)	Consensus not achieved
Denmark	1	21	0	0	13	34 (19.0)	Missing responses not provided
Finland	1	1	2	3	9	15 (8.4)	Missing responses provided
France	4	30	11	6	35	82 (45.8)	Consensus achieved
Germany	5	42	9	7	44	102 (57.0)	Consensus achieved
Greece	2	11	13	3	24	51 (28.5)	Consensus achieved
Hungary	1	3	0	2	1	6 (3.4)	Missing responses provided
Iceland	4	48	17	19	62	146 (81.6)	Consensus not achieved
Ireland	2	46	3	12	48	109 (60.9)	Consensus not achieved
Italy	5	42	12	5	47	106 (59.2)	Consensus achieved
Luxembourg	2	14	8	8	22	52 (29.1)	Consensus achieved
Malta	1	4	0	0	3	7 (3.9)	Missing responses provided
Norway	3	26	9	4	20	59 (32.9)	Consensus achieved
Poland	2	31	1	5	33	70 (39.1)	Consensus achieved
Portugal	1	7	0	2	10	19 (10.6)	Missing responses provided
Romania	1	2	0	3	4	9 (5.0)	Missing responses provided
Russia	7	35	36	17	53	141 (78.8)	Consensus achieved
Serbia	2	14	0	3	18	35 (19.6)	Consensus achieved
Slovakia	4	37	11	6	41	95 (53.0)	Consensus achieved
Slovenia	1	33	0	4	24	61 (34.0)	Missing responses provided
Spain	2	14	6	0	23	43 (24.0)	Consensus achieved
Sweden	3	28	10	12	24	74 (41.3)	Consensus not achieved
Switzerland	2	14	10	2	23	49 (27.3)	Consensus achieved
The Netherlands	2	1	0	0	3	4 (2.2)	Consensus achieved
Turkey	1	35	0	7	38	80 (44.6)	Missing responses provided
United Kingdom	6	26	13	12	31	82 (45.8)	Consensus achieved

*, For further explanation, see text of Supplemental Results. †, Total corresponds to the maximum possible number of the questionnaire responses (i.e., 179).

Table S2. Country-level domain A to D scores in 2015 and 2019, and corresponding summary results.

Country	Domain A		Domain B		Domain C		Domain D	
	**2015**	**2019**	**2015**	**2019**	**2015**	**2019**	**2015**	**2019**
**Austria**	**22**	**30 [38]**	**9**	11	13	11	28	35 [55]
Belgium	28	26 [32]	16	5*	12	13	27	28 [43]
Croatia	4	21* [26]	6	5	13	10	19	30* [40]
Cyprus	11	8 [10]	5	0	16	7	26	25 [33]
Finland	21	33* [42]	10	15	13	15	25	25 [45]
France	18	24 [29]	6	14	11	12	30	29 [50]
Germany	16	22 [25]	4	1	8	12	22	25 [34]
Greece	11	11 [12]	15	6	11	11	15	26* [46]
Hungary	16	20 [24]	19	8*	19	12	26	27 [45]
Italy	8	22* [30]	6	6	10	12	24	25 [35]
Luxembourg	26	24 [28]	12	12	12	11	24	26 [42]
Malta	33	13* [15]	6	5	14	9	25	23 [32]
Norway	12	33* [42]	2	10	19	10	31	31 [50]
Poland	20	20 [22]	4	0	13	12	24	26 [41]
Portugal	18	16 [18]	1	3	10	11	22	20 [27]
Romania	10	9 [9]	0	9	14	9	22	28 [46]
**Russia**	**14**	**14 [14]**	**18**	34	13	11	27	27 [46]
Serbia	1	6 [6]	2	1	11	9	16	20 [37]
Slovakia	19	16 [19]	13	0*	14	11	23	23 [40]
Slovenia	26	19 [23]	4	0	11	10	24	29 [40]
Spain	30	28 [34]	3	5	12	11	22	27 [45]
Switzerland	32	31 [40]	10	6	12	12	26	30 [49]
The Netherlands	41	34 [43]	3	12*	12	16	32	37 [60]
Turkey	7	11 [13]	7	0	12	11	16	21 [35]
United Kingdom	31	28 [37]	8	5	14	20	31	30 [53]
**Mean±SD or Median (IQR)**	**19.0±10.1**	**20.8±8.4 [25.2±11.4]**	**6.0 (3.5-11.0)**	**5.0 (1.0-10.5)**	**12.0 (11.0-14.0)**	**11.0 (10.0-12.0)†**	**24.3±4.5**	**26.9±4.1‡ [42.8±7.8]**
**Coeff. of VAR (All Countries)**	**2.02**	**1.68 [2.28]**					**0.90**	**0.82 [1.56]**

Numbers in brackets are 2019 scores after taking into account responses to Domain A and Domain D questions not included in the 2015 questionnaire. Coeff.of VAR, Coefficient of variation determined as SD / √n, where n=number of countries. *, Changes from 2015 to 2019 correspond to ≥10 points. †, P=0.02vs. 2015. ‡, P=0.04 vs. 2015.

Table S3. Detailed results on the primary outcome and subscores and their components in 25 countries (as defined in Methods of the main paper).

DOMAIN A – Practices / decisions related to EOL care (n=25 countries)	2019	2015	P-value
**Do-not-attempt CPR**	**6.0 (2.0-8.0)**	**3.0 (1.0-6.5)**	NA
Advance Directives	4.0 (2.0-7.0)	2.0 (0.0-6.5)	NA
Advance Care Planning	3.0 (0.0-7.0)	NA	NA
Terminal Analgesia / Sedation	1.0 (1.0-2.0)	2.0 (1.0-2.0)	NA
Termination-of-Resuscitation Protocols	1.0 (0.0-4.0)	2.0 (1.0-4.5)	NA
Treatment Limitation	4.0 (3.0-5.0)	4.0 (2.5-4.5)	NA
Euthanasia / Physician-Assisted Suicide	0.0 (0.0-0.0)	0.0 (0.0-0.0)	NA
Transportation Practices	2.0 (1.0-2.0)	2.0 (1.0-2.0)	NA

**EOL Practices Subscore (2019 vs. 2015) ***	**18.2±7.5**	**16.4±9.2**	**0.45**
**EOL Practices Subscore 2019 †**	**21.6±10.4**	**NA**	**NA**
Family participation in EOL Decisions (Adults & Children)	2.0 (0.5-2.0)	2.0 (1.0-2.0)	NA
Shared Decision Making	2.0 (1.5-2.0)	NA	NA
**EOL Decisions (Adults & Children) Subscore (2019 vs. 2015) ***	**2.0 (0.5-2.0)**	**2.0 (1.0-2.0)**	**>0.99**
**EOL Decisions (Adults & Children) Subscore 2019 †**	**4.0 (2.0-4.0)**	NA	NA
**Family Presence during Resuscitation Subscore (2019 vs. 2015) ***	**1.0 (0.0-2.0)**	**0.0 (0.0-2.0)**	**0.90**
**DOMAIN A Score (2019 vs. 2015) ***<br>**Coefficient of variation**	**20.8±8.4**<br>**1.68**	**19.0±10.1**<br>**2.02**	**0.51**
**DOMAIN A Score 2019 †**<br>**Coefficient of variation**	**25.8±11.8**<br>**2.36**	NA	NA
**DOMAIN B – Access to Best Resuscitation and Postresuscitation Care**	**2019**	**2015**	**P-value**
Determinants of Access to Best Resuscitation Care (OHCA)	2.0 (0.0-3.0)	2.0 (1.0-3.5)	0.26
Determinants of Access to Best Resuscitation Care (IHCA)	2.0 (1.0-3.5)	2.0 (1.0-3.0)	0.79
Determinants of Access to Best Postresuscitation Care	2.0 (0.0-4.0)	2.0 (1.5-4.5)	0.38
**DOMAIN B Score (2019 vs. 2015) ***	**5.0 (1.0-10.5)**	**6.0 (3.5-11.0)**	**0.41**
**DOMAIN C – Death Diagnosis and Organ Donation**	**2019**	**2015**	**P-value**
Allowed to Diagnose Death	2.0 (2.0-2.0)	2.0 (2.0-2.0)	NA
Allowed to Diagnose Death after 20 min Asystole without Reversible Cause (OHCA)	2.0 (2.0-2.0)	2.0 (2.0-2.0)	NA
Brain Death or Cardiorespiratory Death Criteria for Death Diagnosis	5.0 (4.0-5.0)	5.0 (4.0-5.0)	NA
**Death Diagnosis Subscore (2019 vs. 2015) ***	**9.0 (8.0-9.0)**	**9.0 (8.0-10.0)**	**0.19**
**Organ Donation Subscore (2019 vs. 2015) ***	**3.0 (2.0-3.0)**	**4.0 (2.0-4.0)**	**0.03**
**DOMAIN C Score (2019 vs. 2015) ***	**11.0 (10.0-12.0)**	**12.0 (11.0-14.0)**	**0.02**

**DOMAIN D – Emergency Care Organization**	**2019**	**2015**	**P-value**
Access to Care in Rural Areas	3.0 (2.0-3.0)	3.0 (1.5-3.5)	NA
Access to Care in Urban Areas	3.0 (3.0-4.0)	3.0 (2.5-4.0)	NA
Access to Care In-hospital	2.0 (1.0-2.0)	2.0 (2.0-2.0)	NA
**Access to Emergency Care Subscore (2019 vs. 2015) ***	**8.0 (7.0-9.0)**	**8.0 (6.0-9.0)**	**0.66**
Legally Allowed to Defibrillate	6.0 (5.5-6.0)	6.0 (4.5-6.0)	NA
Availability of Defibrillation	6.0 (4.0-70)	5.0 (3.0-70)	NA
Automated External Defibrillator Data Availability	0.0 (0.0-1.0)	NA	NA
Public Access Defibrillation	4.0 (3.0-5.0)	3.0 (1.5-4.0)	NA
**Defibrillation Subscore (2019 vs. 2015) ***	**15.0 (13.0-17.0)**	**13.0 (10.5-15.0)**	**0.01**
**Defibrillation Subscore 2019 †**	**15.0 (13.5-17.0)**	**NA**	**NA**
Rescuer Alert System	1.0 (0.0-1.0)	NA	NA
Dispatcher Assisted CPR	4.0 (4.0-6.0)	NA	NA
Ambulance Level of Care	4.0 (3.0-4.0)	3.0 (1.5-4.0)	NA
Traumatic Cardiac Arrest Protocol	3.0 (2.0-4.0)	NA	NA
**Level of Out-of-hospital Care Subscore (2019 vs. 2015) ***	**4.0 (3.0-4.0)**	**3.0 (1.5-4.0)**	**0.008**
**Level of Out-of-hospital Care Subscore 2019 †**	**12.0 (10.5-13.5)**	NA	NA
Inhospital Rapid Response Teams	1.0 (0.0-1.0)	NA	NA
CPR Debriefing, Audit, and Feedback	0.0 (0.0-1.5)	0.0 (0.0-1.5)	NA
CPR Training	0.0 (0.0-0.0)	0.0 (0.0-0.0)	NA
**Organization of In-hospital Emergency Services Subscore (2019 vs. 2015) ***	**1.0 (0.0-2.0)**	**1.0 (0.0-2.0)**	**0.84**
**Organization of In-hospital Emergency Services Subscore 2019 †**	**2.0 (0.5-2.5)**	NA	NA
**Registry Reporting of Cardiac Arrest Subscore (2019 vs. 2015) ***	**1.0 (1.0-2.0)**	**0.0 (0.0-1.0)**	**0.002**
Ethics Education Programs	3.6±2.2	NA	NA
Mandatory CPR Training	0.0 (0.0-2.0)	NA	NA
**Education Subscore 2019 †**	**4.0 (2.0-5.0)**	**NA**	**NA**
**Research and Informed Consent Subscore 2019 †**	**0.0 (0.0-1.0)**	**NA**	**NA**
**DOMAIN D Score (2019 vs. 2015) ***<br>**Coefficient of variation**	**26.9±4.1**<br>**0.82**	**24.3±4.5**<br>**0.90**	**0.04**
**DOMAIN D Score 2019 †**<br>**Coefficient of variation**	**42.8±7.8**<br>**1.56**	**NA**	**NA**

EOL, end-of-life; CPR, cardiopulmonary resuscitation; NA, not applicable; OHCA, out-of-hospital cardiac arrest; IHCA, in-hospital cardiac arrest. Domain scores and subscores, and

corresponding P-values are highlighted in bold. For the purpose of uniform presentation, data are presented as median (interquartile range) whenever at least one of the reported 2019 or 2015 variables exhibited a skewed distribution of its values; otherwise (i.e. in the presence of variables with always normal distributions of their values), data are presented as mean±SD; the coefficient of variation was determined as SD / √n (n=number of participating countries).*, Scores determined according to variables included in both the 2019 and 2015 questionnaires. †, Scores determined according to all variables included in the 2019 questionnaire; these variables were those included in the 2015 questionnaire plus additional ones, as further detailed in Table 1 of the main paper.

Table S4. Detailed results on the secondary outcome and subscores and their components in countries with ″low″ 2015 domain total scores (as defined in Methods of the main paper).

**DOMAIN A – Practices / decisions related to EOL care (n=13 countries)**	**2019**	**2015**	**P-value**
Do-not-attempt CPR	3.0 (0.0-7.0)	1.0 (0.0-3.5)	NA
Advance Directives	2.0 (0.5-5.0)	0.0 (0.0-2.0)	NA
Advance Care Planning	2.4±2.9	NA	NA
Terminal Analgesia / Sedation	1.0 (0.5-2.0)	2.0 (0.0-2.0)	NA
Termination-of-Resuscitation Protocols	1.9±2.0	1.8±1.5	NA
Treatment Limitation	3.4±1.6	2.2±1.8	NA
Euthanasia / Physician-Assisted Suicide	0.0 (0.0-0.0)	NA	NA
Transportation Practices	2.0 (1.5-2.0)	2.0 (1.0-2.0)	NA
**EOL Practices Subscore (2019 vs. 2015) ***	**14.8±6.9**	**9.4±5.2**	**0.04**
**EOL Practices Subscore 2019 †**	**17.2±9.5**	**NA**	**NA**
Family participation in EOL Decisions (Adults & Children)	2.0 (0.0-2.0)	2.0 (0.0-2.0)	NA
Shared Decision Making	2.0 (0.0-2.0)	NA	NA
**EOL Decisions (Adults & Children) Subscore (2019 vs. 2015) ***	**2.0 (0.5-2.0)**	**2.0 (0.0-2.0)**	**>0.99**
**EOL Decisions (Adults & Children) Subscore 2019 †**	**4.0 (0.0-4.0)**	NA	NA
**Family Presence during Resuscitation Subscore (2019 vs. 2015) ***	**0.0 (0.0-1.5)**	**0.0 (0.0-1.0)**	**0.047**
**DOMAIN A Score (2019 vs. 2015) *<br>Coefficient of variation**	**16.7±7.8<br>2.16**	**11.2±5.3<br>1.45**	**0.047**
**DOMAIN A Score 2019 †<br>Coefficient of variation**	**20.2±10.9<br>3.02**	NA	NA
**DOMAIN B – Access to Best Resuscitation and Postresuscitation Care (n=15 countries)**	**2019**	**2015**	**P-value**
Determinants of Access to Best Resuscitation Care (OHCA)	1.0 (0.0-3.0)	1.0 (1.0-2.0)	0.59
Determinants of Access to Best Resuscitation Care (IHCA)	1.0 (0.0-3.0)	1.0 (1.0-2.0)	0.78
Determinants of Access to Best Postresuscitation Care	2.0 (0.0-3.0)	2.0 (0.0-2.0)	0.60
**DOMAIN B Score (2019 vs. 2015) ***	**4.7±4.7**	**3.9±2.1**	**0.55**
**DOMAIN C – Death Diagnosis and Organ Donation (n=13 countries)**	**2019**	**2015**	**P-value**
Allowed to Diagnose Death	2.0 (2.0-2.0)	2.0 (2.0-2.0)	NA
Allowed to Diagnose Death after 20 min Asystole without Reversible Cause (OHCA)	2.0 (2.0-2.0)	2.0 (2.0-2.0)	NA
Brain Death or Cardiorespiratory Death Criteria for Death Diagnosis	5.0 (4.0-5.0)	4.0 (3.5-5.0)	NA
**Death Diagnosis Subscore (2019 vs. 2015) ***	**9.0 (8.0-9.0)**	**8.0 (7.5-9.0)**	**0.24**
**Organ Donation Subscore (2019 vs. 2015) ***	**3.0 (2.0-3.0)**	**3.0 (2.0-4.0)**	**0.82**
**DOMAIN C Score (2019 vs. 2015) ***	**11.0 (11.0-12.0)**	**11.0 (10.5-12.0)**	**0.63**

**DOMAIN D – Emergency Care Organization (n=9 countries)**	**2019**	**2015**	**P-value**
Access to Care in Rural Areas	2.6±0.9	2.6±1.2	NA
Access to Care in Urban Areas	3.0 (3.0-4.0)	3.0 (1.0-4.0)	NA
Access to Care In-hospital	1.0 (1.0-2.0)	2.0 (1.5-2.0)	NA
**Access to Emergency Care Subscore (2019 vs. 2015) ***	**7.4±1.5**	**6.9±2.3**	**0.55**
Legally Allowed to Defibrillate	6.0 (4.0-6.0)	5.0 (4.0-6.0)	NA
Availability of Defibrillation	5.0±1.8	2.7±1.3	NA
Automated External Defibrillator Data Availability	0.0 (0.0-1.0)	NA	NA
Public Access Defibrillation	2.4±1.1	1.8±1.2	NA
**Defibrillation Subscore (2019 vs. 2015) ***	**12.3±3.9**	**9.2±2.8**	**0.07**
**Defibrillation Subscore 2019 †**	**12.7±3.6**	**NA**	**NA**
Rescuer Alert System	0.0 (0.0-1.0)	NA	NA

Dispatcher Assisted CPR	4.8±1.4	NA	NA
Ambulance Level of Care	4.0 (3.5-4.0)	2.0 (1.0-3.0)	NA
Traumatic Cardiac Arrest Protocol	2.0 (2.0-4.0)	NA	NA
**Level of Out-of-hospital Care Subscore (2019 vs. 2015) ***	**4.0 (3.5-4.0)**	**2.0 (1.0-3.0)**	**0.002**
**Level of Out-of-hospital Care Subscore 2019 †**	**12.0 (11.5-12.5)**	NA	NA
Inhospital Rapid Response Teams	1.0 (0.0-1.0)	NA	NA
CPR Debriefing, Audit, and Feedback	1.0 (0.0-1.5)	0.0 (0.0-2.0)	NA
CPR Training	0.0 (0.0-0.0)	0.0 (0.0-0.0)	NA
**Organization of In-hospital Emergency Services Subscore (2019 vs. 2015) ***	**1.0 (0.0-2.0)**	**0.0 (0.0-2.0)**	**0.60**
**Organization of In-hospital Emergency Services Subscore 2019 †**	**1.6±1.4**	NA	NA
**Registry Reporting of Cardiac Arrest Subscore (2019 vs. 2015) ***	**1.0 (1.0-1.5)**	**0.0 (0.0-0.5)**	**0.01**
Ethics Education Programs	3.6±2.6	NA	NA
Mandatory CPR Training	0.0 (0.0-1.0)	NA	NA
**Education Subscore 2019 †**	**3.8±3.1**	**NA**	**NA**
**Research and Informed Consent Subscore 2019 †**	**0.0 (0.0-1.0)**	**NA**	**NA**
**DOMAIN D Score (2019 vs. 2015) ***<br>**Coefficient of variation**	**24.4±3.6**<br>**1.20**	**19.7±3.2**<br>**1.07**	**0.009**
**DOMAIN D Score 2019 †**<br>**Coefficient of variation**	**38.9±6.4**<br>**2.13**	**NA**	**NA**

EOL, end-of-life; CPR, cardiopulmonary resuscitation; NA, not applicable; OHCA, out-of-hospital cardiac arrest; IHCA, in-hospital cardiac arrest. Domain total scores and subscores, and corresponding P-values are highlighted in bold. For the purpose of uniform presentation, data are presented as median (interquartile range) whenever at least one of the reported 2019 or 2015 variables exhibited a skewed distribution of its values; otherwise (i.e. in the presence of variables with always normal distributions of their values), data are presented as mean±SD; the coefficient of variation was determined as SD / √n (n=number of participating countries). *, Scores determined according to variables included in both the 2019 and 2015 questionnaires. †, Scores determined according to all variables included in the 2019 questionnaire; these variables were those included in the 2015 questionnaire plus additional ones, as further detailed in Table 1 of the main paper.

Table S5. Results on domain total scores and subscores and their components in countries with ″high″ 2015 domain total scores (as defined in Methods of the main paper).

**DOMAIN A – – Practices / decisions related to EOL care (n=12 countries)**	**2019**	**2015**	**P-value**
Do-not-attempt CPR	6.4±2.4	5.9±2.6	NA
Advance Directives	6.5 (3.3-8.0)	6.0 (3.3-8.0)	NA
Advance Care Planning	4.6±3.2	NA	NA
Terminal Analgesia / Sedation	1.5 (0.3-2.0)	2.0 (2.0-2.0)	NA
Termination-of-Resuscitation Protocols	1.5 (0.3-4.8)	3.5 (2.0-5.0)	NA
Treatment Limitation	4.0 (4.0-5.0)	4.0 (4.0-5.0)	NA
Euthanasia / Physician-Assisted Suicide	0.0 (0.0-1.8)	0.0 (0.0-3.3)	NA
Transportation Practices	2.0 (1.0-2.0)	2.0 (2.0-2.0)	NA
**EOL Practices Subscore (2019 vs. 2015) ***	**21.9±6.4**	**23.9±6.0**	**0.44**
**EOL Practices Subscore 2019 †**	**26.5±9.4**	**NA**	**NA**
Family participation in EOL Decisions (Adults & Children)	2.0 (2.0-2.0)	2.0 (0.0-2.0)	NA
Shared Decision Making	2.0 (2.0-2.0)	NA	NA
**EOL Decisions (Adults & Children) Subscore (2019 vs. 2015) ***	**2.0 (2.0-2.0)**	**2.0 (0.0-2.0)**	**>0.99**
**EOL Decisions (Adults & Children) Subscore 2019 †**	**4.0 (4.0-4.0)**	NA	NA
**Family Presence during Resuscitation Subscore (2019 vs. 2015) ***	**1.4±1.2**	**1.6±1.2**	**0.73**
**DOMAIN A Total Score (2019 vs. 2015) ***<br>**Coefficient of variation**	**25.2±6.8**<br>**1.96**	**27.4±6.4**<br>**1.85**	**0.42**
**DOMAIN A Total Score 2019 †**<br>**Coefficient of variation**	**31.8±9.9**<br>**2.86**	NA	NA
**DOMAIN B – Access to Best Resuscitation and Postresuscitation Care (n=10 countries)**	**2019**	**2015**	**P-value**
Determinants of Access to Best Resuscitation Care (OHCA)	3.0 (1.8-3.5)	4.0 (3.0-5.3)	0.046
Determinants of Access to Best Resuscitation Care (IHCA)	2.5 (1.8-4.5)	3.0 (2.8-6.0)	0.35
Determinants of Access to Best Postresuscitation Care	3.4±3.4	4.8±4.3	0.24

**DOMAIN B Score (2019 vs. 2015) ***<br>**Coefficient of variation**	**10.2±9.4**<br>**2.97**	**13.0±3.9**<br>**1.23**	**0.39**

DOMAIN C – Death Diagnosis and Organ Donation (n=12 countries)	2019	2015	P-value
Allowed to Diagnose Death	2.0 (2.0-2.8)	2.5 (2.0-4.0)	NA
Allowed to Diagnose Death after 20 min Asystole without Reversible Cause (OHCA)	2.0 (2.0-2.8)	2.0 (2.0-3.8)	NA
Brain Death or Cardiorespiratory Death Criteria for Death Diagnosis	4.0 (3.0-5.0)	5.0 (5.0-5.8)	NA
**Death Diagnosis Subscore (2019 vs. 2015) ***	**8.5 (7.0-9.0)**	**10.0 (9.0-12.0)**	**0.009**
**Organ Donation Subscore (2019 vs. 2015) ***	**3.0 (2.0-3.0)**	**4.0 (2.5-4.0)**	**0.003**
**DOMAIN C Score (2019 vs. 2015) ***	**11.0 (9.3-12.0)**	**14.0 (13.0-15.5)**	**0.002**

DOMAIN D – Emergency Care Organization (n=16 countries)	2019	2015	P-value
Access to Care in Rural Areas	3.0 (2.3-3.8)	3.0 (1.3-3.0)	NA
Access to Care in Urban Areas	3.5 (3.0-4.0)	3.5 (3.0-4.0)	NA
Access to Care In-hospital	2.0 (1.0-2.0)	2.0 (2.0-2.0)	NA
**Access to Emergency Care Subscore (2019 vs. 2015) ***	**8.0 (7.0-9.0)**	**8.0 (7.3-9.0)**	**0.94**
Legally Allowed to Defibrillate	6.0 (6.0-6.0)	6.0 (6.0-6.0)	NA
Availability of Defibrillation	6.0 (4.3-7.0)	6.0 (5.0-7.0)	NA
Automated External Defibrillator Data Availability	0.0 (0.0-1.0)	NA	NA
Public Access Defibrillation	4.5(3.0-5.0)	3.5 (2.0-4.0)	NA
**Defibrillation Subscore (2019 vs. 2015) ***	**15.7±2.3**	**14.1±2.0**	**0.051**
**Defibrillation Subscore 2019 †**	**16.0±2.3**	**NA**	**NA**
Rescuer Alert System	1.0 (0.3-1.0)	NA	NA
Dispatcher Assisted CPR	4.0 (4.0-6.0)	NA	NA
Ambulance Level of Care	4.0 (3.0-4.0)	3.0 (2.3-4.0)	NA
Traumatic Cardiac Arrest Protocol	3.2±1.7	NA	NA
**Level of Out-of-hospital Care Subscore (2019 vs. 2015) ***	**3.5 (1.3-4.0)**	**3.0 (2.3-4.0)**	**0.44**
**Level of Out-of-hospital Care Subscore 2019 †**	**3.3±1.1**	NA	NA
Inhospital Rapid Response Teams	1.0 (0.0-1.0)	NA	NA
CPR Debriefing, Audit, and Feedback	1.0 (0.0-1.8)	0.5 (0.0-1.8)	NA
CPR Training	0.0 (0.0-0.8)	0.0 (0.0-0.8)	NA
**Organization of In-hospital Emergency Services Subscore (2019 vs. 2015) ***	**1.0 (0.0-1.8)**	**1.0 (0.0-2.0)**	**0.82**
**Organization of In-hospital Emergency Services Subscore 2019 †**	**1.8±1.5**	NA	NA
**Registry Reporting of Cardiac Arrest Subscore (2019 vs. 2015) ***	**1.5 (1.0-2.0)**	**1.0 (0.0-1.0)**	**0.045**
Ethics Education Programs	3.9±2.0	NA	NA
Mandatory CPR Training	2.0 (0.0-2.8)	NA	NA
**Education Subscore 2019 †**	**6.0 (3.3-7.0)**	**NA**	**NA**
**Research and Informed Consent Subscore 2019 †**	**0.0 (0.0-1.0)**	**NA**	**NA**
**DOMAIN D Score (2019 vs. 2015) ***<br>**Coefficient of variation**	**28.3±3.7**<br>**0.93**	**26.9±2.8**<br>**0.70**	**0.23**
**DOMAIN D Score 2019 †**<br>**Coefficient of variation**	**44.9±7.8**<br>**1.95**	**NA**	**NA**

EOL, end-of-life; CPR, cardiopulmonary resuscitation; NA, not applicable; OHCA, out-of-hospital cardiac arrest; IHCA, in-hospital cardiac arrest. Domain total scores and subscores, and corresponding P-values are highlighted in bold. For the purpose of uniform presentation, data are presented as median (interquartile range) whenever at least one of the reported 2019 or 2015 variables exhibited a skewed distribution of its values; otherwise (i.e. in the presence of variables with always normal distributions of their values), data are presented as mean±SD; the coefficient of variation was determined as SD / √n (n=number of participating countries). *, Scores determined according to variables included in both the 2019 and 2015 questionnaires. †, Scores determined according to all variables included in the 2019 questionnaire; these variables were those included in the 2015 questionnaire plus additional ones, as further detailed in Table 1 of the main paper.

Table S6. Changes in responses to the 2019 questionnaire due to the coronavirus disease-19 (COVID-19) pandemic.

Country	CHANGES PERTAINING TO DOMAINS A TO D CAUSED BY THE SARS-COV-2 PANDEMIC
Austria	No change reported
Belgium	No change reported

Country	CHANGES PERTAINING TO DOMAINS A TO D CAUSED BY THE SARS-COV-2 PANDEMIC
Croatia	FPDR not allowed during the pandemic; change in Domain A score: -3
Cyprus	Post-CPR audit initiated; change in Domain D score: +1
Finland	FPDR for children not allowed during the pandemic; change in Domain A score: -1
France	No change reported
Germany	OHCA: applications to alert trained lay rescuers and/or first responders deactivated; change in Domain D score: -1
Greece	No change reported
Hungary	No change reported
Italy	Reduced number of transplantations (Domain C – no score change); delays in response to emergency calls; dispatcher-assisted CPR available only for home CPR due to the COVID-19 lockdown (Domain D – no score change)
Luxembourg	IHCA: high-risk presentation now affects access to best resuscitation care (Domain B total score: +1); uncertainty about continuation of OHCA and IHCA registries (Domain D score: -2)
Malta	Registry reporting of IHCA (Domain D score +1); consultants now obliged to state DNACPR status of admitted patients
Norway	No response provided
Poland	No change reported
Portugal	No change reported
Romania	Access to resuscitation care affected by comorbidity (OHCA) and age (IHCA); change in Domain B score: +2
Russia	No change reported
Serbia	No change reported
Slovakia	No response provided
Slovenia	Slovenian guidelines for first responders/BLS, medical dispatch services, and out-of-hospital, in-hospital ALS were modified. CPR hands-on courses stopped; (Domain D – no score change)
Spain	No change reported
Switzerland	No change reported
The Netherlands	No change reported
Turkey	No change reported
United Kingdom	No change reported

SARS-COV-2, severe acute respiratory syndrome coronavirus 2; FPDR, family presence during resuscitation; CPR, cardiopulmonary resuscitation; OHCA, out-of-hospital cardiac arrest; IHCA, in-hospital cardiac arrest; DNACPR, do-not-attempt CPR: BLS, basic life support; ALS, advanced life support.

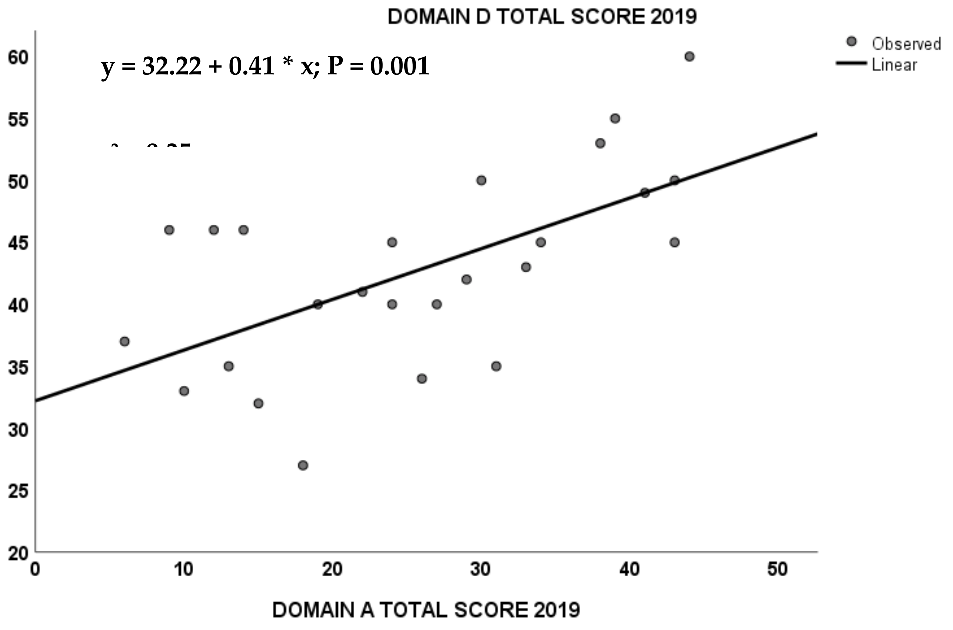

Figure S1A

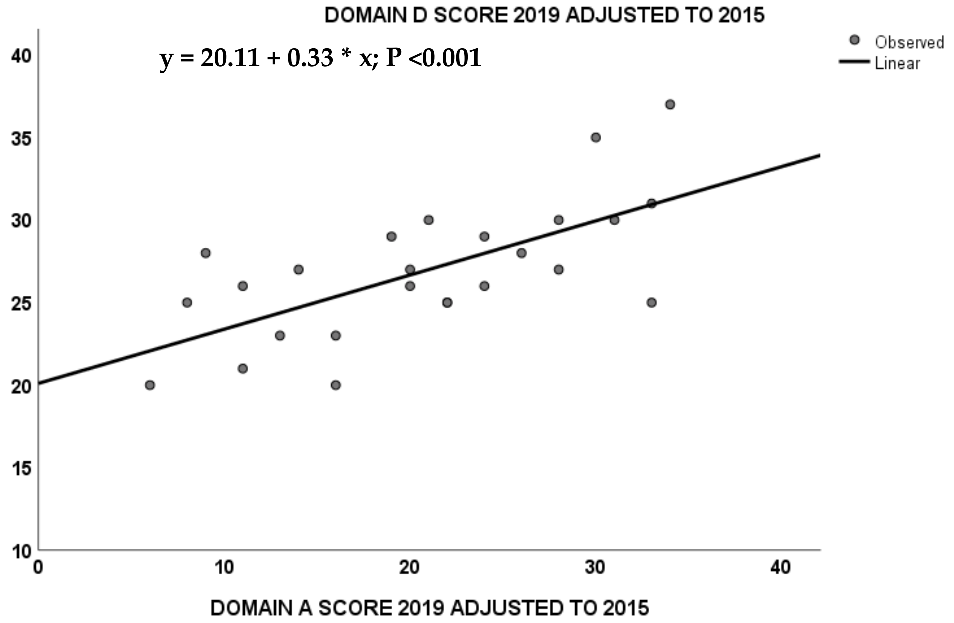

Figure S1B

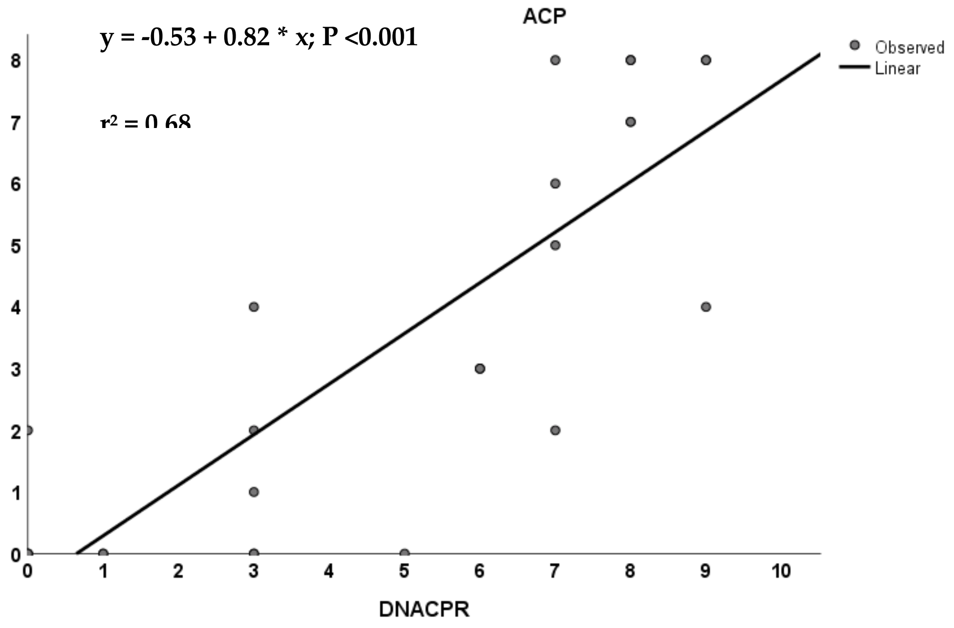

Figure S1C

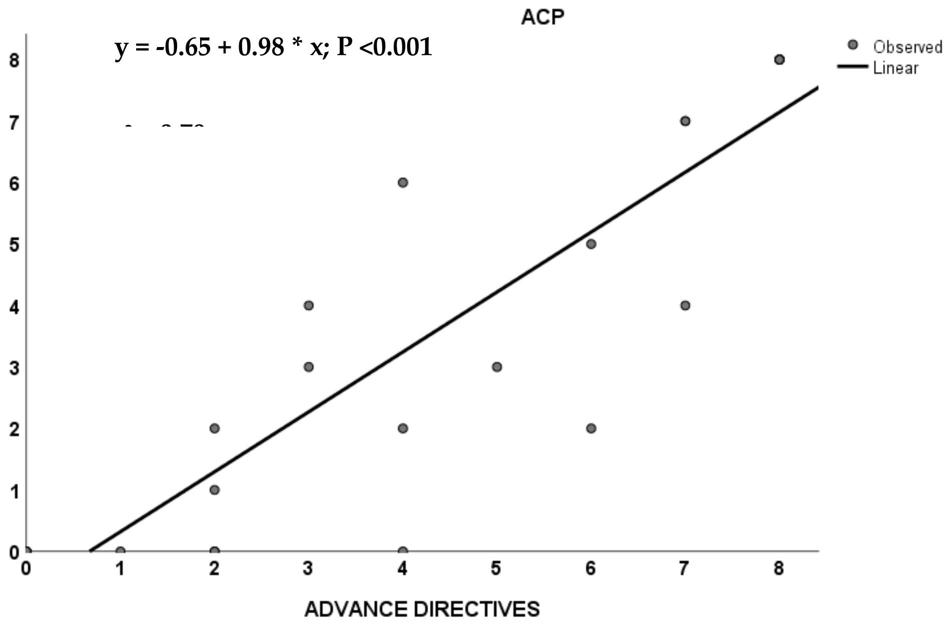

Figure S1D

Figure S1. Graphs of linear relationships between 2019 domain A and domain D scores with (**A**) and without (**B**) the questions included only in the 2019 survey (see also above and Table 1 of the main paper); Graphs of linear relationships between 2019 do-not-attempt cardiopulmonary resuscitation (DNACPR) and advance care planning (ACP) scores (**C**), and between 2019 advance directives and ACP scores (**D**).
